# Assessing the impact of Djdi on the growth, nutrient uptake, and secondary metabolite production in *Eruca vesicaria* (L.) Cav.

**DOI:** 10.1186/s12870-026-08864-5

**Published:** 2026-05-23

**Authors:** Esraa E. Ammar, Basma M. Khalaf, Mohamed A. A. Shaalan, Ahmed Gelany, Doaa E. Elsherif

**Affiliations:** 1 Botany Department, Faculty of Science, Tanta University, Tanta, 31527 Egypt; 2 Agricultural Natural Resources Department, Faculty of Agriculture and Natural Resources, Aswan University, Aswan, Egypt; 3 Geology Department, Faculty of Science, Luxor University, Luxor, Egypt

**Keywords:** Djdi, Soil Fertility, *Eruca vesicaria* (L.) Cav., Secondary metabolites

## Abstract

**Background:**

This pioneering research aims to assess Djdi Ocher (Khuzam Shale), a naturally occurring Pharaonic fertilizer from Southern Egypt, as a unique sustainable soil amendment. The study focuses on blending Djdi with sandy clay soil (CS) in various ratios to evaluate its effects on the growth and development of Eruca vesicaria.

**Results:**

In a pot experiment, *E. vesicaria* seeds were germinated in three different Djdi-to-soil ratios: (1:1; Djdi: CS1), (1:2; Djdi: CS2), and (1:3; Djdi: CS3). After 15 days, seedlings were harvested for comprehensive analysis. XRD analysis characterized Djdi as a sandy loam enriched with minor and major oxides, particularly iron oxides. Soil analysis revealed that Djdi: CS2 and Djdi: CS3 treatments recorded the highest sum of anions (CO_3_^-2^, HCO_3_^-^, Cl^-^, and SO_4_^-2^) and cations (N^+^, P^+ 5^, Ca^+ 2^, Na^+^, K^+^, Fe^+ 2^, and Mg^+ 2^). In plants, the Djdi: CS3 treatment significantly enhanced the accumulation of N^+^, P^+ 5^, K^+^, Ca^+ 2^, and Fe^+ 2^. Furthermore, Djdi: CS3 achieved the highest germination rates, mineral absorption, and growth parameters, alongside an increase in secondary metabolites (phenolics and flavonoids). Notably, stress biomarkers (MDA and H_2_O_2_) were significantly elevated in all Djdi-treated samples. RT-PCR analysis confirmed the up-regulation of key secondary metabolite genes, including chalcone synthase (CHS), phenylalanine ammonia-lyase (PAL), flavonol synthase (FLS), and chalcone isomerase (CHI), particularly in the Djdi: CS3 group.

**Conclusion:**

The study concludes that the Djdi: CS3 (1:3) ratio serves as a novel, eco-friendly fertilizer that improves soil fertility, promotes plant growth, and boosts bioactive constituents. These findings align with Sustainable Development Goals (SDGs) by enhancing soil productivity for subsequent crop cycles.

**Graphical Abstract:**

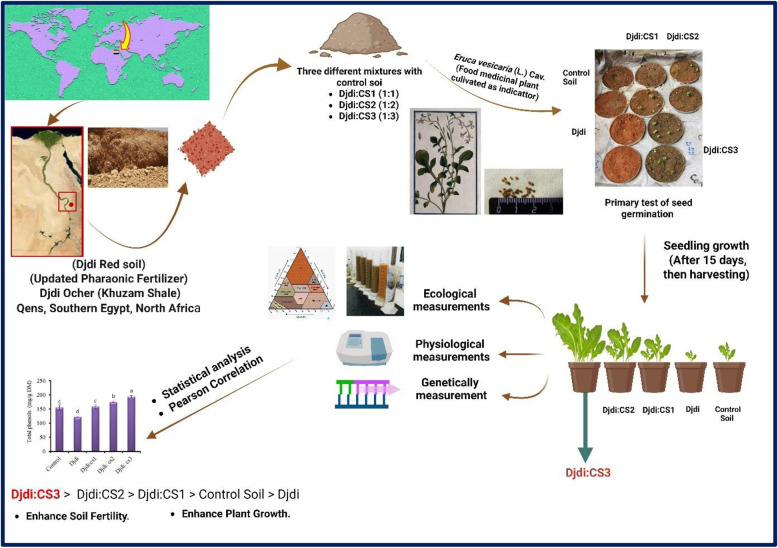

**Supplementary Information:**

The online version contains supplementary material available at 10.1186/s12870-026-08864-5.

## Highlights

1. Evaluation of Djdi Ocher (Khuzam Shale) as an updated Eco-friendly Pharaonic Bio-fertilizer.

2. Assessing the impact of blending Djdi with sandy clay soil in different ratios on the growth of *E. vesicaria*.

3. Application of Djdi: CS3 (1:3) fertilizer can enhance the growth and bioactive compounds of *E. vesicaria*, while also improving soil fertility.

4. Djdi: CS3 (1:3) is a novel fertilizer aligns with sustainable development goals (SDGs) and improves soil fertility for subsequent crop cycles.

**Supplementary Information:**

The online version contains supplementary material available at 10.1186/s12870-026-08864-5.

## Introduction

The use of natural soil rich in macronutrients or micronutrients as a natural fertilizer is essential for replenishing nutrient-deficient soils [1]. This approach serves as a sustainable fertilization method that aligns with the 2030 Sustainable Development Goals, including land conservation and climate action [2, 3]. The excessive use of chemical fertilizers has become one of the major threats to global agricultural sustainability. Although they enhance crop productivity in the short term, chemical fertilizers often cause serious long-term damage to soil health, water resources, and the environment [4–6]. Continuous application leads to soil acidification, loss of microbial diversity, accumulation of toxic residues, and contamination of groundwater with nitrates and phosphates, ultimately reducing soil fertility and contributing to climate change [7]. Moreover, the energy-intensive production of synthetic fertilizers releases large amounts of greenhouse gases, aggravating global warming and ecosystem imbalance [2, 3, 5, 8].

In contrast, natural or bio-based fertilizers have emerged as sustainable alternatives capable of maintaining soil fertility while minimizing environmental harm. These materials, derived from organic or mineral sources, restore the balance of essential nutrients, enhance soil structure, and support beneficial microbial activity [8–10]. They improve the soil’s physical and chemical properties, promote nutrient retention, and release macro- and micronutrients gradually, ensuring long-term soil productivity. The adoption of such natural amendments directly supports several of the United Nations’ Sustainable Development Goals (SDGs), including land conservation, zero hunger, and climate action [3, 6, 9, 11]. Common examples include organic fertilizers such as compost, farmyard manure, green manure, and vermicompost, which enrich the soil with carbon, nitrogen, and beneficial microorganisms; biofertilizers containing living microbes such as *Rhizobium*, *Azotobacter*, *Azospirillum*, and phosphate-solubilizing bacteria, which fix atmospheric nitrogen and enhance nutrient availability; and mineral-based fertilizers of natural origin such as rock phosphate, dolomite, gypsum, and volcanic ash, which supply vital macro- and micronutrients like calcium, phosphorus, and magnesium [6, 8, 12]. Among the promising mineral-based fertilizers, Djdi Ocher (Khuzam Shale) from southern Egypt represents an ancient yet underexplored resource. Known historically as “red ochre” or “Daffah” in some Arabic dialects, Djdi is rich in iron oxides and trace elements essential for plant growth. Archaeological and geological evidence indicates that red ochre was extensively used in Pharaonic Egypt for pigmentation, dyeing, and other applications, highlighting its mineral richness and long-standing environmental relevance. Geochemical analyses of the Khuzam Formation reveal high concentrations of Fe₂O₃, CaO, MgO, SiO₂, and K₂O, minerals known to enhance soil structure and fertility [12–14]. The Khuzam shale is rich in basic plant nutrients, including essential minerals important for the plant life cycle (e.g., Fe, K, P, Ca) and growth-promoting elements (e.g., Na, Si, Co) [12, 15]. This nutritional structure is well systemized on the periodic table and can be further categorized either based on how much is found in plant tissue as either a macro or micronutrient, or based on their ionic charge as either cations or anions. Therefore, utilizing Djdi as a natural mineral fertilizer could serve as a sustainable approach to revitalize nutrient-depleted soils in arid environments and reduce dependence on chemical fertilizers [12].

A vital vegetable crop, *E. vesicaria* belongs to the Brassicaceae family [16]. It is a valuable source of important metabolites and has a high market value [17, 18]. Because of its rich nutritional content and wide variety of health-promoting bioactive components, its leaves are frequently eaten as salad [19]. Heterocyclics, sulfur-containing organics, phenolics (23.60%), terpenoids, organic esters, and silyl compounds are among the six categories of phytochemicals found in *E. vesicaria* leaves [20]. Numerous medicinal advantages of *E. vesicaria*, such as phytochemicals, have been shown, including antibacterial, anti-inflammatory, anticancer, antioxidant, and antigenotoxic assets [21]. Based on these important characteristics, *E. vesicaria* potential can be used to increase its cultivation and generate those useful substances [18, 19]. Accordingly, this research is considered a novel point dating back to Pharaonic mythological origins around the possibility of using of Djdi as a sustainable, eco-friendly fertilizer in small quantities. This study aims to evaluate the fertilization efficiency of Djdi–clay soil mixtures at different ratios on the growth, nutrient uptake, and metabolic activity of *E. vesicaria*., a medicinal and edible plant valued for its nutritional and therapeutic properties. The research also seeks to highlight Djdi’s potential as an eco-friendly bio-fertilizer consistent with global sustainable agriculture goals.

## Materials and methods

### Study area

**Fig. 1:**
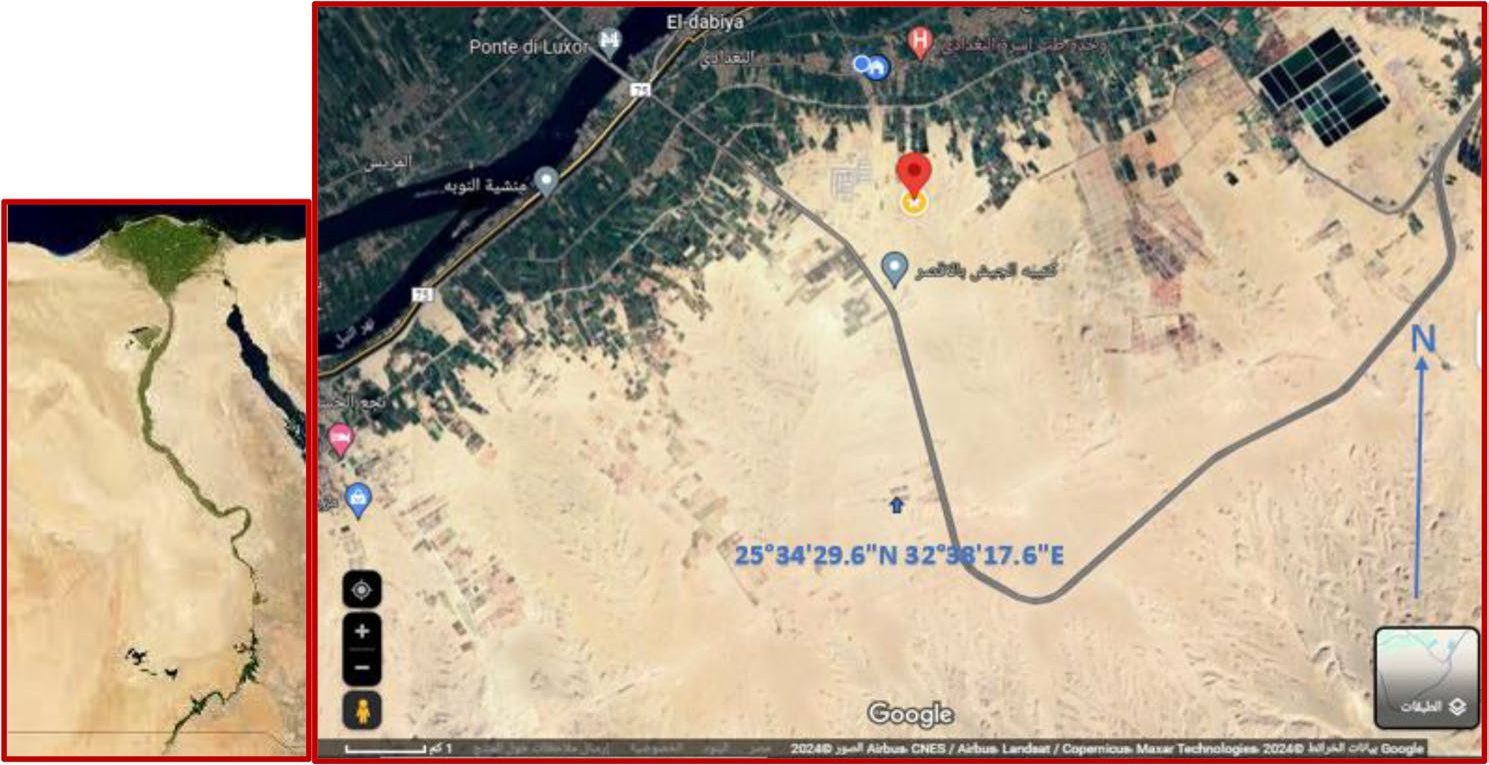
A map of the Khuzam Shale old quarry from Google Earth (https://earth.google.com)

Djdi is still mined locally in a series of old quarries, one of which is at 25°34’29.6"N 32°38’17.6"E, although the use of Djdi in the past is obviously evident; these sites are now abandoned. The formation of these sediments is geologically credited to the Khuzam Formation who is the oldest of four rock formations during the Miocene era, making the base of the Qena Lake, Egypt, Northern East Africa. The given formation is distinguished by a stratum of brown shale, which is crowned by the masses of conglomerates and sands (Fig. 1).

### Experimental preparation

Djdi were collected from the study area (Djdi) (0–50 cm) from the topsoil profile utilizing a shovel made of stainless steel, subsequent for eradicating of the superficial debris, Didi areas are considered as public deserts in Egypt which is located around the Qena.

Lake, Egypt, Northern East Africa. It is available from collection in small amounts without causing harms according to Egyptian Constitution 2024, 453/1969 and 143/1981. In the future, intensive use may be organized through state-sanctioned mining licenses and land reclamation projects. They do not strictly prohibit intensive use, provided it is conducted under formal governmental oversight and environmental impact assessments.

The control clay soil samples were collected from the author own land, in Tanta, Nile Delta, Egypt (30° 40’ N, 30° 56’ E). Treatments were prepared as followings; Djdi: pure Djdi soil, Control: Clay Delta Soil, Djdj: CS1 (1 Djdi: 1 control), Djdj: CS2 (1 Djdi : 2 control) and Djdj: CS3 (1 Djdi : 3 control). Fifty seeds of *Eruca vesicaria* L. Cav. (Sementi Da Orto, Milan, Italy) were surface-sterilised in 10% commercial bleach [5% (w/v) NaOCl] for 10 min, followed by three rinses with distilled water. Seeds were cultivated for 15 days during summer 2024 under dark conditions at room temperature in transparent pots (1000 ml) at the greenhouse of the Faculty of Science, Tanta University, Egypt [22]. The species was identified by Dr. Esraa E. Ammar, and a voucher specimen (No. 300, 15/12/2017) was deposited in the Tanta University Herbarium (TANE) [16].

#### Mineral Evaluation of Djdi as a novel mineral fertilizer

Mineral Analysis by XRD for 3 different samples of collected Djdi; 1 gram of clay sample from each soil was placed in a sample holder of the XRD machine to determine the X-ray patterns. XRD patterns were acquired with a D8 Advance (Bruker) X-ray diffractometer with Cu Kα radiation at 40 kV and 40 mA, at a scanning rate of 2˚ 2θ min − 1 across a 2θ range of 0˚ to 90˚. Subsequently, the peaks were analysed for the mineralogical phase using “Eva” software. The mineral analysis was determined using software by comparing the spectra of pure minerals.

### Soil texture and physical parameters

Sieve-Pipette Method (SPM) was used for the determination of clay, sand, and silt percentages in each treatment. A pipette was used to withdraw a fixed volume of soil-water suspension at a predetermined depth and time interval. The samples were subsequently dried and evaporated until a consistent weight was achieved. The particle size distribution was then transformed into a recognizable texture class using the soil texture triangle. All soil samples were examined using SPM for their particle size distributions (PSDs). Following the extraction of rock pieces, air-dried samples underwent pre-treatment and complete dispersion, after which the soil suspension was filtered through a 100-mm screen to isolate the 100–2000 mm fraction. The soil particles smaller than 100 mm were transported to a 1-L cylinder for future pipette analysis. This segment was categorized into four size classes: < 2 mm, 2–20 mm, 20–53 mm, and 53–100 mm. The first three classes were directly measured using a pipette, while the final class was indirectly computed by deducting the proportions of the other three size classes, including the one obtained through sieving, from 100%. Three replicates were conducted for each sample, and the mean particle size distribution (PSD) was recorded [23, 24]. Soil-water extracts at a 1:5 (w/v) ratio were prepared to determine electrical conductivity (EC) and pH values using a conductivity meter (60 Sensor Operating Instruction Corning) and a glass electrode pH meter (Model HI 2211), respectively.

### Mineral soil analysis

HNO_3_: concentrated HCl) (v/v) on a hot plate for 3 h, subsequently followed Atomic Absorption Spectrometry (AAS) was employed to analyse soil extracts derived from 5 g of air-dried soil samples, utilizing 2.5% (v/v) glacial acetic acid to evaluate the availability of nutrients such as phosphate, potassium, and calcium. Total nitrogen was measured using the Micro-Kjeldahl method. To evaluate the total concentrations of Fe, a 0.5–1 g sub-sample was obtained from each composite sample and digested in 20 ml of freshly prepared aqua regia (1:3 concentrated by evaporation and analysis of total heavy metal concentrations. After digestion, samples were permitted to cool to ambient temperature, generally yielding a colourless or, on occasion, pink hue. The molybdenum blue method was utilized for phosphorus analysis using a spectrophotometer (CECIL CE 1021). Potassium and calcium were quantified utilising a flame photometer (CORNING M410). Total anions were quantified, encompassing N, P, K, Ca, Mg, Na, and Fe [25]. In addition, total anions were calculated, including CO₃, HCO₃, Cl, and So_4_ [25]. The methodologies for soil investigation were delineated by [25–27]. The apparatus setup and operational settings were executed in accordance with the manufacturer’s specs.

### Mineral plant analysis

Minerals were mined from 0.5 to 1 g dry weight samples via 20 ml of a nitric acid and hydrogen peroxide solution (3:2 v/v) until a clear hue was observed. A segment of the sample (0.5 g DW) was allocated for macronutrient examination, whereas (1 g DW) was designated for heavy metal investigation. Total nitrogen was quantified with the Micro-Kjeldahl technique. The Molybdenum blue procedure was employed to ascertain total phosphorus, utilising a spectrophotometer (CECIL CE 1021). Potassium and calcium were quantified utilising a flame photometer (CORNING M410), whereas iron at 239.562 nm was assessed by inductively coupled plasma (ICP) (Perkin Elmer, Optima 7000 DV). All of these techniques were delineated [25]. The equipment setup and functional settings were executed in accordance with the manufacturer’s specifications. The amounts of P^+ 5^, N^+^, K^+^, Ca^+^, and Fe^+^ concentrations are reported as mg/L.

### Assessment of growth patterns and secondary metabolite production

After a thorough washing, the seedlings (15 days old) were gathered, and their fresh weights were estimated. Fresh seedlings were dried for three days at 50 °C to determine their dry weight. The ethanolic extract of the shoot was used to determine the quantities of total phenolics and flavonoids, according to [28, 29]. Fresh leaves were pulverized into a fine powder after being dried. At room temperature, tissues (0.1 and 0.5 g) were homogenized and incubated for 30 min in 70% acetone, and then centrifuged at 4 xg. Phenolic content was quantified using Folin Ciocalteu reagent. The extract was mixed with Folin-Ciocalteu reagent (1:1) (V: V). Sodium carbonate solution 7.5% was added (2 V) after 5 min. After 2 h, the absorbance was measured at 725 nm.

100 µl of the same extract was combined with 4 cc of distilled water for the flavonoid assay, and 0.3 mL of 5% sodium nitrite was added after that. Five minutes later, 0.3 ml of 10% aluminum chloride was added. 2 ml of 1 M sodium hydroxide was added to the mixture after 5 min. The absorbance was quantified relative to a blank at 510 nm. Catechin was utilized for the calibration curve.

### Evaluation of stress biomarkers

Malondialdehyde (MDA) and H_2_O_2_ concentrations were measured using [30] and [31], respectively. To determine H_2_O_2_, 0.1 g of fresh leaves was extracted in 0.1% trichloroacetic acid (TCA). The absorbance was measured at 390 nm after the plant extract was combined with phosphate buffer (pH 7.0) and KI_2_. A leaf was extracted in a 5% (w/v) TCA solution to assess MDA. After 20 min of heating, the sample extract and 0.67% (w/v) TBA were cooled. The absorbance of the sample was measured at two wavelengths (532 and 600 nm).

### Genetic measurements

Quantitation of relative expression levels of the exact key genes in the RNA extract was carried out using the RNeasy Plant Mini Kit according to the instructions (Qiagen). cDNA was created using the High-Capacity cDNA Reverse Transcription Kit (Applied Biosystems). Quantitative RT-PCR was performed exactly [32]. The relative gene expression level was analyzed with the 2−∆∆CT method [33] using *GAPDH* as the reference gene (Table 1).

**Table 1:** Sequences of designated primers utilized in qRT-PCR

Gene	Abbreviation	Direction	Sequences (5^’^-3^’^)
Reference gene	*GAPDH*	F	AAGGTTATCAACGACAGGTTTG
		R	ATACCCTTAAGCTTGCCTTCTG
Phenylalanine ammonia lyase	*PAL*	F	GCAAGGAAAGCCCGAGTTTAC
		R	GGACCTTTTTGGCTACTTGGC
Chalcone synthase	*CHS*	F	CCCGATTACTATTTTCGGATCAC
		R	CGAGTGAATCAAGGTGAGTGTC
Chalcone isomerase	*CHI*	F	TGGTGGCCTAGACAACGATGAGTT
		R	TCACACTCCCAACTTGGTTTCCCT
Flavonol synthase	*FLS*	F	TTAAAGGAAGGTCTCGGTGGCGAA
		R	TCATTGGTGACGATGAGTGCGAGT

### Statistical Examination

All outcomes presented are means of three biological replicates, each consisting of at least 4 plants. Analysis of variance of the data was conducted using the XLSTAT software (version 2014.5.03). The treatment means were analyzed using Tukey’s test (*p* < 0.05).

## Results

### Mineral evaluation of Djdi as a novel mineral fertilizer

Based on the provided chemical composition and XRD analysis, Khuzam shale is a complex, multi-mineral material with a diverse elemental profile (Table 2). A key characteristic of this shale is its fine particle size, with an average of 72 nm. Chemically, it is characterized by high concentrations of CaO (17.0%) and SiO₂ (23.8%), alongside significant amounts of SO₃ (5.3%), Na₂O (9.9%), Fe₂O₃ (6.8%), Al₂O₃ (6.7%), and MgO (5.7%). This composition is directly reflected in its mineralogy, which is dominated by Calcite and Dolomite (accounting for the high CaO and MgO), Quartzite (from SiO₂), Anhydrite (the source of SO₃), and Halite (from Na₂O and Cl).

**Table 2:** The result of XRD of three samples of Khuzam shale

	CaO	MgO	SiO_2_	TiO_2_	Al_2_O_3_	Fe_2_O_3_	Na_2_O	K_2_O	P_2_O_5_	Cl	SO_3_
**Khuzam shale**	17.0 ± 3.9^(b)^	5.7 ± 1.15^(d)^	23.8 ± 0.47^(e)^	1.0 ± 0.15^(f)^	6.7 ± 0.28^(d)^	6.8 ± 0.24^(d)^	9.9 ± 5.4^(c)^	0.6 ± 0.16^(g)^	0.6 ± 0.26^(g)^	5.3 ± 2.8^(de)^	0.4 ± 0.06^(h)^

### Soil texture and physical parameters

**Fig. 2:**
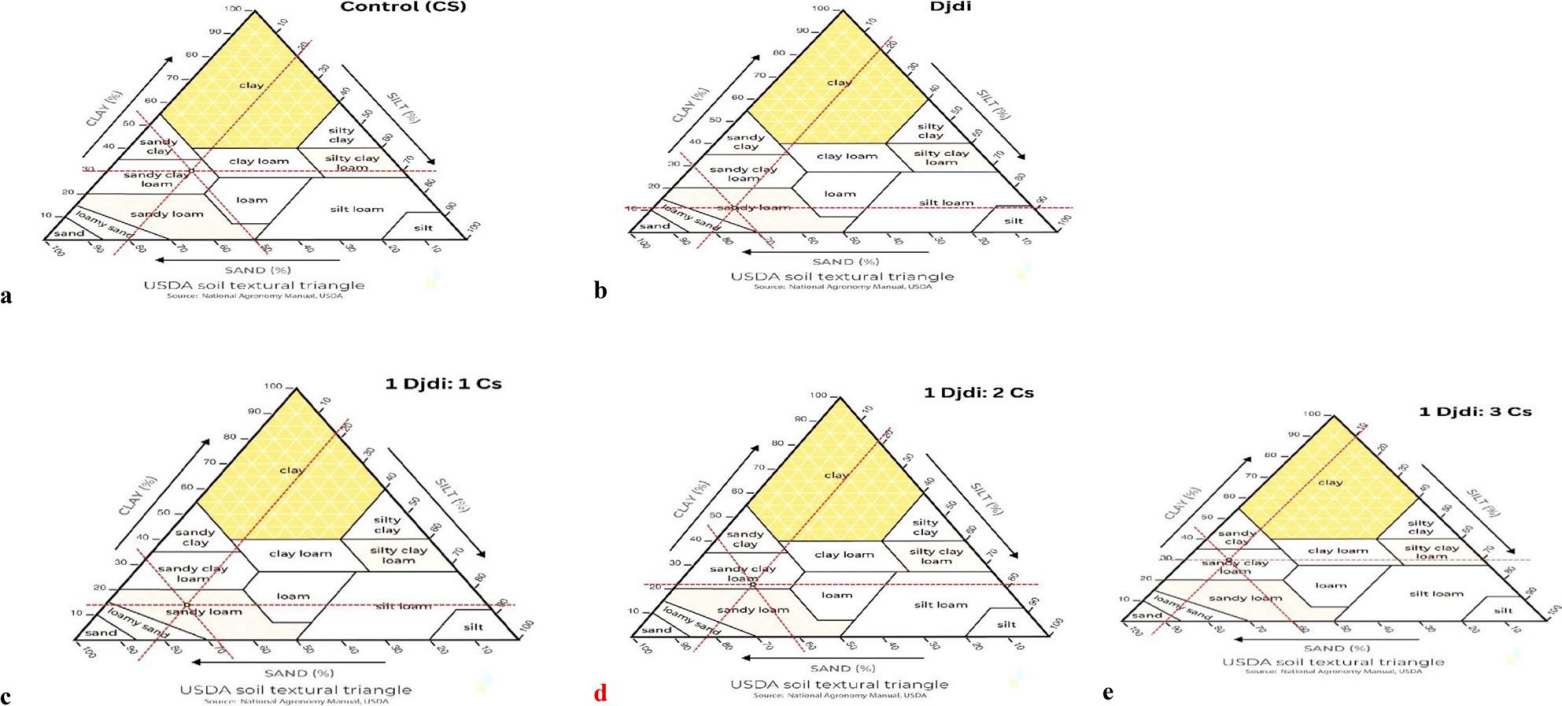
Soil texture. **(a)** control (treated soil) (CS), **(b)** Djdi Ocher (Khuzam Shale) (Djdi), Different concentrations of treated clay soil with Djdi **(c)** Djdj: CS1, **(d)** Djdj: CS2 and **(e)** Djdj: CS3

Djdj is a red sandy loam soil (Fig. 2a). After mixture of Djdi (sand = 70%, clay = 12%, silt = 18%) with different concentrations of treated soil (sand = 50%, clay = 30%, silt = 20%); Djdj: CS1 (sand = 68%, clay = 18%, silt = 14%) became sandy loam, while Djdj: CS2 (sand = 62%, clay = 22%, silt = 16%) and Djdj: CS3 (sand = 60%, clay = 3 0%, silt = 10%) became sandy clay loam (Fig. 2b, c, and **d**).

About soil pH mean; while treated control soil was 7.89 and Djdj was 7.86. Djdi: CS1was decreased till 7.81, and Djdi: CS2 was 7.72, while Djdi: CS3 increased slightly till 7.88 (Table 3**)**. About soil EC mean, while the treated control soil was 1.99 and Djdj was 15.18. Djdi: CS1was 7.05, and Djdi: CS2 was 7.86, while Djdi: CS3 was 4.1 (Table 3**)**.

**Table 3:** Soil measurements of control and other treatments

Soil measurements	Control	Djdi	Djdi: CS1	Djdi: CS2	Djdi: CS3
pH means	8 ± 0.006 ^(a)^	7.9 ± 0.006 ^(c)^	7.8 ± 0.006 ^(d)^	7.7 ± 0.006 ^(e)^	7.9 ± 0.006 ^(b)^
EC (ms/cm)	2 ± 0.6 ^(e)^	15.2 ± 0.006 ^(a)^	7.1 ± 0.006 (c)	7.9 ± 0.6 ^(b)^	4.1 ± 0.06 ^(d)^
Total anions (mg/L)	28.3 ± 0.06 ^(e)^	130.1 ± 2.9 ^(a)^	73.4 ± 1.2 ^(c)^	87.8 ± 1.2 ^(b)^	47.3 ± 1.2 ^(d)^
Co_3_^-2^ (mg/L)	3.1 × 10^4^±0.6 ^(e)^	19.7 × 10^4^±0.9 ^(a)^	11.02 × 10^4^±0.6 ^(d)^	17.2 × 10^4^±0.1 ^(b)^	15.9 × 10^4^±0.06 ^(c)^
HCo_3_^-^ (mg/L)	4 ± 0.3 ^(c)^	10 ± 03^(a)^	7 ± 06 ^(a)^	9 ± 07^(b)^	5 ± 0.4 ^(c)^
So_4_^-2^ (mg/L)	2 ± 0.06 ^(a)^	10.06 ± 0.03 ^(a)^	2.4 ± 0.06 ^(c)^	3.8 ± 0.06 ^(b)^	2.3 ± 0.06 ^(c)^
Cl^-^ (mg/L)	22.5 ± 0.06 ^(e)^	110 ± 0.6^(a)^	62 ± 0.6 ^(c)^	77 ± 0.6 ^(b)^	40 ± 0.6^(d)^
Total cations (mg/L)	28.3 ± 1.1^(e)^	130.1 ± 5.2 ^(a)^	73.4±0.0.06 ^(c)^	87.8 ± 1.2 ^(b)^	47.3 ± 1.2 ^(d)^
K^+^ (mg/L)	0.6 ± 0.006^(c)^	0.5 ± 0.008^(d)^	0.9 ± 0.008^(a)^	0.8 ± 0.003^(b)^	0.3 ± 0.009^(e)^
N^+^ (mg/L)	0.5 × 10^3^±0.006 (d)	0.01 × 10^3^±0.0 (e)	0.8 × 10^3^±0.006(c)	1.3 × 10^3^±0.006 (a)	1.2 × 10^3^±0.006 (b)
P^+ 5^ ppm (mg/L)	5.1 ± 0.0006 ^(c)^	3.8 ± 0.0006 ^(e)^	5.4 ± 0.0006 ^(d)^	11.3 ± 0.0006 ^(a)^	9.9 ± 0.0006 ^(b)^
Fe^+ 2^ (mg/L)	50 ± 0.06 ^(e)^	216.7 ± 0.06 ^(a)^	107.6 ± 0.06 ^(b)^	89.5 ± 0.06 ^(c)^	70.5 ± 0.06 ^(d)^
Ca^+ 2^ (mg/L)	6 ± 0.6 ^(e)^	40 ± 0.6 ^(a)^	18 ± 0.6 ^(c)^	22 ± 0.6 ^(b)^	8 ± 0.6 ^(d)^
Na^+^ (mg/L)	12.7 ± 0.09 ^(e)^	45.6 ± 0.06 ^(a)^	40.6 ± 0.08 ^(c)^	45 ± 0.6 ^(b)^	30 ± 0.6 ^(d)^
Mg^+ 2^ (mg/L)	7 ± 0.6 ^(e)^	44 ± 0.6 ^(a)^	14 ± 0.6 ^(c)^	20 ± 0.6 ^(b)^	9 ± 0.6 ^(d)^

### Mineral soil analysis

The mineral analysis revealed significant differences in ionic composition between the treatments. The Djdi exhibited the highest overall mineral content, with total anions and cations reaching 130.1 mg/L, significantly greater than the control (28.3 mg/L). This was driven by substantial increases in chloride (Cl), carbonate (Co₃), and cations like sodium (Na), calcium (Ca), and magnesium (Mg). The application of Djdj: CS treatments (CS1, CS2, CS3) effectively mitigated this salt accumulation in a dose-dependent manner. Furthermore, the CS treatments, particularly CS2 and CS3, had a notable positive impact on plant nutrition, significantly increasing the concentrations of key nutrients such as phosphorus (P), nitrogen (N), and potassium (K) compared to both the control and the stressed Djdi group. For instance, phosphorus in Djdi: CS2 (11.3 ppm) was more than double that of the control. Iron (Fe) content was highest in the Djdi group, but was substantially reduced by all CS treatments.

### Mineral plant analysis

**Fig. 3:**
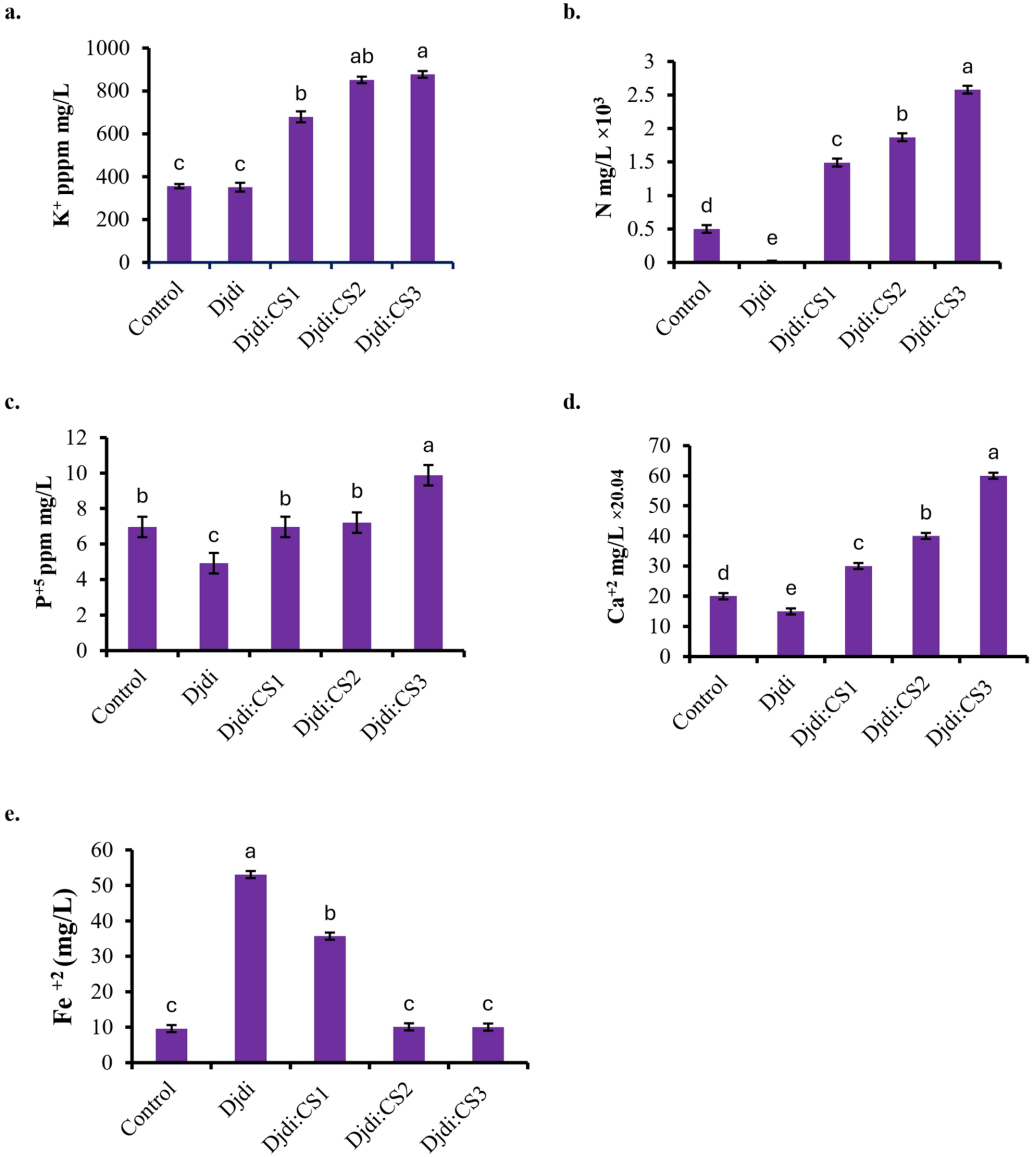
According to Atomic Absorption Spectrometry analysis of essential minerals of plant samples, **a** k^+^(ppm mg/L), **b** N %, **c** P^+ 5^ (ppm mg/L), **d** Ca^+ 2^ (mg/L), and **e** Fe^+ 2^ (mg/L)

The application of Djdj: CS treatments significantly influenced nutrient availability, demonstrating a clear dose-dependent effect. For K and Ca, the Control treatment showed the lowest concentration. The Djdj: CS treatments substantially increased K and Ca availability, with the Djdi: CS3 treatment proving most effective, resulting in the highest K levels. A similar positive trend was observed for phosphorus (P) availability. Among the treatments, Djdi: CS2 and Djdi: CS3 were the most effective, significantly enhancing P concentration compared to the Control and Djdj: CS variants. In contrast, the pattern for Fe was inversely related. The data indicate that the Control maintained the highest Fe levels, while all Djdj: CS treatments resulted in a marked reduction (Fig. 3**)**.

### Seed germination and seedlings growth criteria

**Fig. 4:**
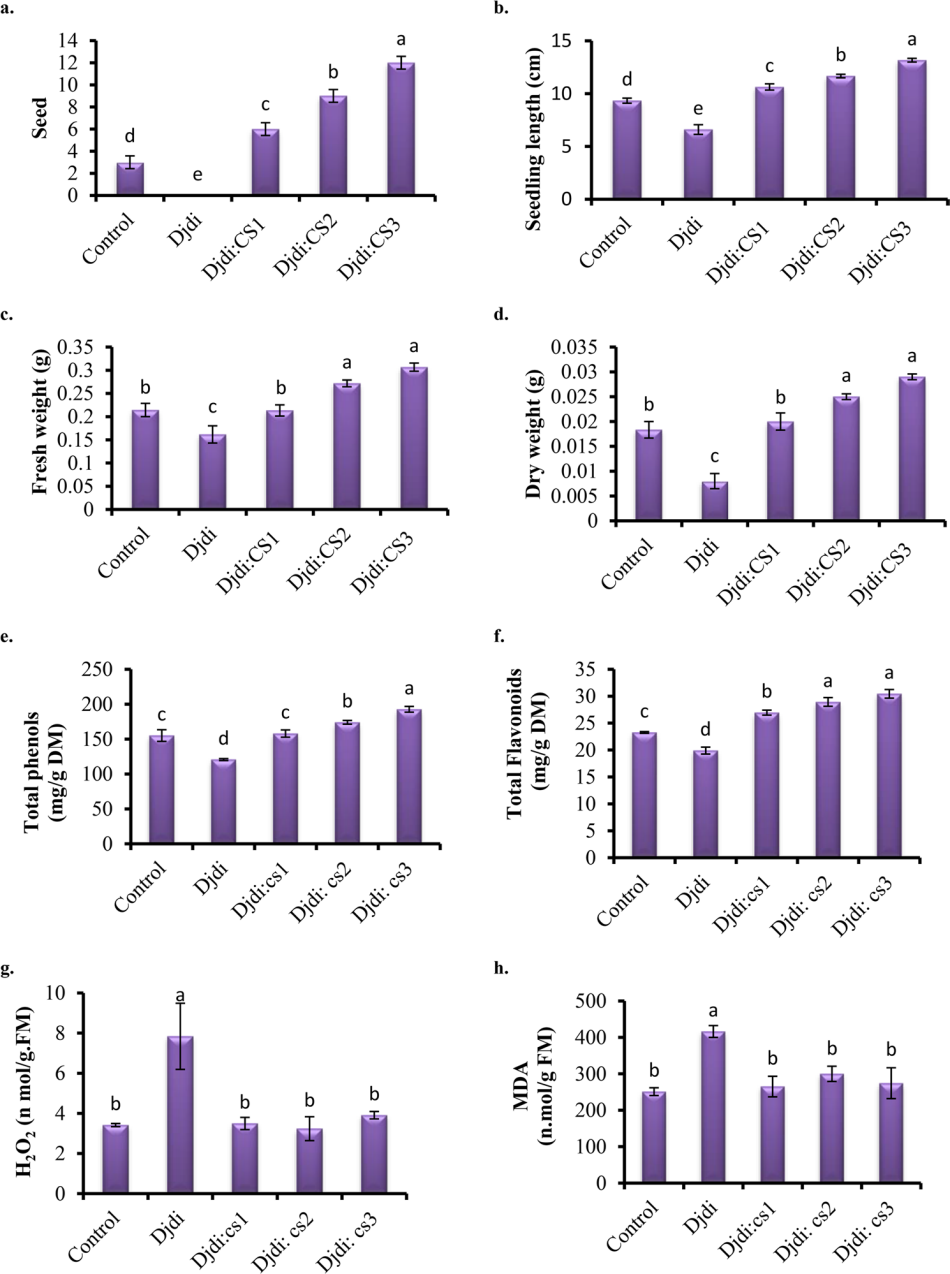
Effect of different concentrations of clay soil either singly or mixed with Djdj soil on (**a**) seed germination, (**b**) seedlings length (cm), and (**c**) fresh and (**d**) dry weights (mg), (**e**) total phenolics, (**f**) total flavonoids contents, (**g**) hydrogen peroxide (H_2_O_2_), and (**h**) malondialdehyde (MDA) of *Eruca vesicaria* seedling

Seeds of *E. vesicaria* were surface sterilized with 10% sodium hypochloride solution for 10 min and then rinsed with distilled water. The primary test of seed germination of 15 seeds of *E. vesicaria* with different treatments after 4 days showed that germination rates of 20% for the control, 0% for Djdj, 40% for Djdj: CS1, 60% for Djdj: CS2, and 80% for Djdj: CS3. When Djdj was applied alone, the seedling length, fresh weight, and dry weight decreased by 29.28%, 24.57%, and 56.36%, respectively, relative to the control. Application of Djdj to different concentrations of clay soil significantly increased the seedling length by 13.9, 23.1 and 41.2% at Djdj: CS1, Djdj: CS2 and Djdj: CS3, correspondingly in relation to the control. Concerning seedlings fresh weight, the magnitude of increase in seedlings fresh and dry weight at mixed Djdj with 2 and 3 of clay soil treatment was 26.75 and 43.08%, 36.39 and 58.21%, respectively, compared with the control (Fig. 4**)**.

### Phenolic and flavonoid content

The data indicated that the total phenolic and total flavonoids content gradually increases with increasing the concentration of clay soil mixed with Djdj specially at Djdj: CS3 treatment reaching 24.24% and 30.81%, respectively, as compared with the control (Fig. 4). However, application of Djdj soil decreases the total phenols content achieving 22.2 0% and 14.48%, respectively, relative to the control.

### Content of H_2_O_2_ and MDA

Based on the provided data, Djdt induced significant oxidative stress in the plants, as evidenced by a substantial increase in H₂O₂ and MDA levels by 129.24% and 65.74% compared to the well-watered Control. The application of Djdj: CS effectively mitigated this oxidative damage (H₂O₂ and MDA) in a dose-dependent manner (Fig. 4).

### Genetic measurement

#### Gene expression

**Fig. 5:**
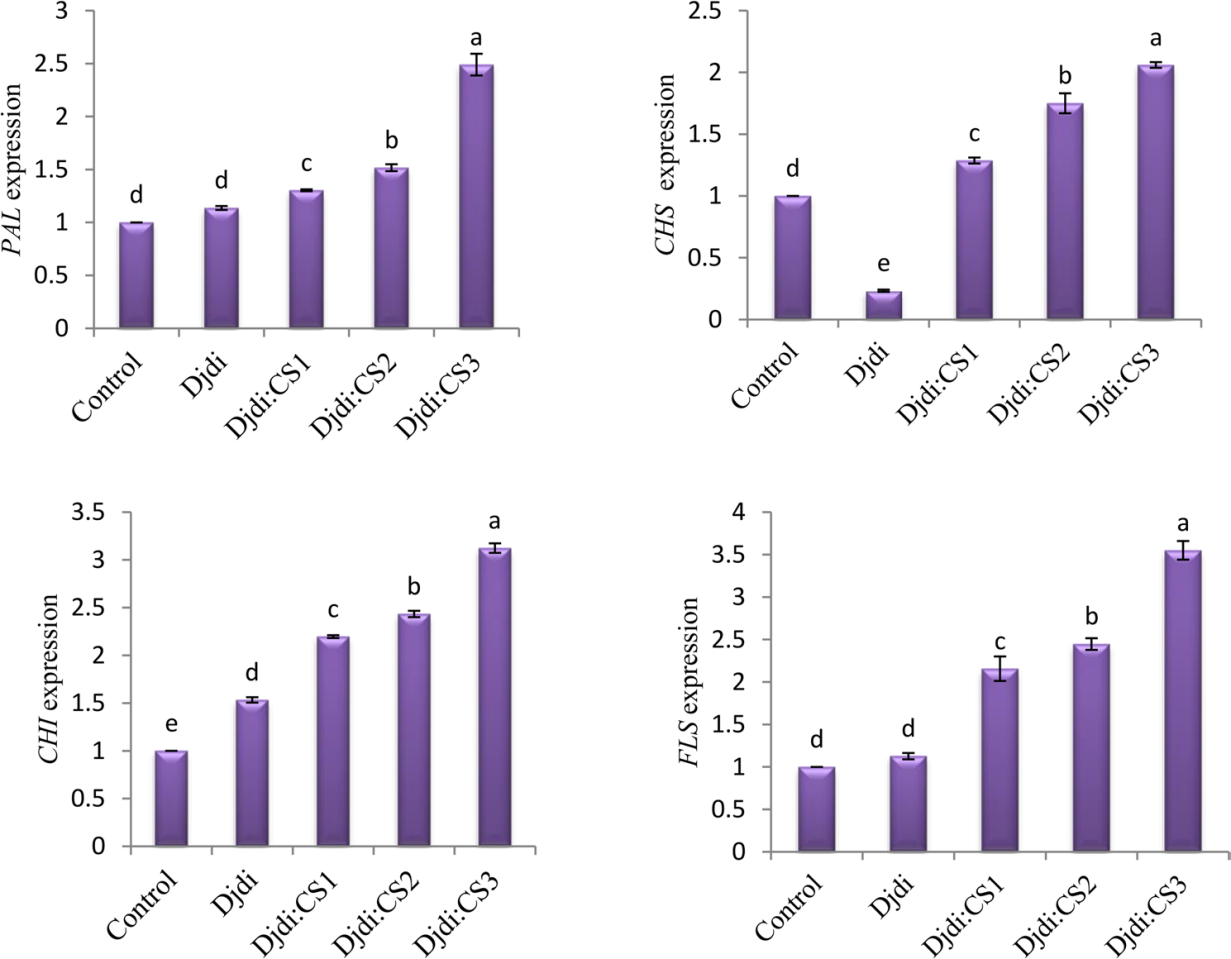
The effect of different Djdi and CS treatment on *PAL*,* CHS*,* CHI*, and *FLS* genes expressions of *Eruca vesicaria* seedlings

The study looked into gene expression of a number of important enzymes involved in secondary metabolites, such as CHI, CHS, FLS, and PAL. In comparison to the control, the outcomes demonstrated that plants treated with Djdi: CS had much greater transcript levels and increased expression of several genes. In particular, the highest increases in CHI, CHS, FLS, and PAL expression were 2.12, 1.06, 2.55, and 1.46-fold, respectively, with the Djdi: CS3 treatment dosage. However, during Djdi treatment, PAL and FLS gene expression showed a non-significant increase relative to the control, while CHS expression was lower than in the control (Fig. 5).

### Pearson correlation coefficient

**Fig. 6:**
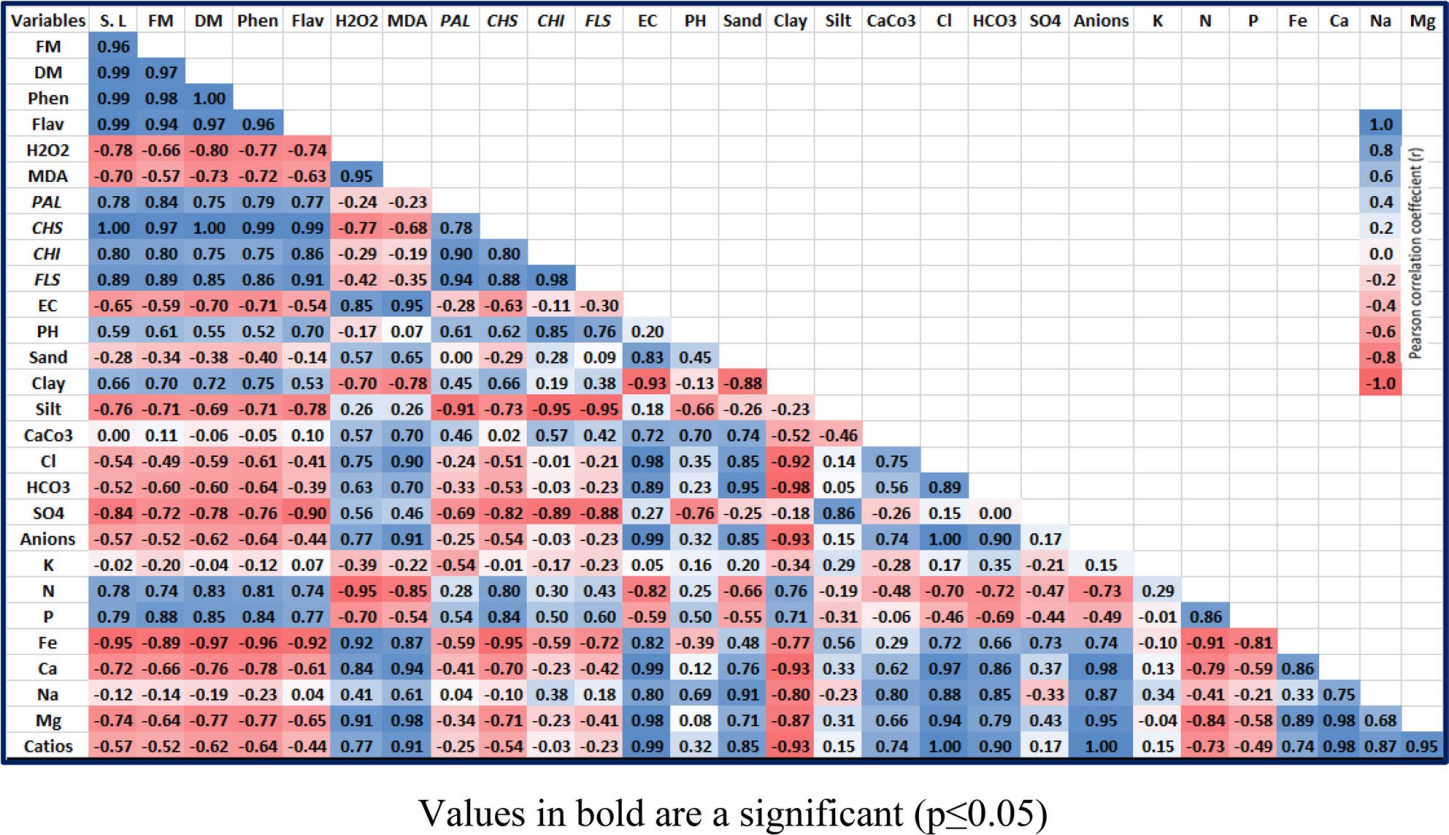
Heat Map of Pearson Correlation Coefficient analyses examine the connections molecular, ecological, and physiological characteristics detected in treatments with Djdi: CS. Values in bold are a significant (*p* ≤ 0.05)

Using the Djdi: CS application, the correlations between ecological, physiological, and molecular features seen in different treatments were examined using Pearson’s simple correlation (Fig. 6). The results showed a substantial positive relationship between clay percentage in soil, N and P soil contents, and soil pH, in addition to phenolic and flavonoid contents, and growth parameters. Increased expression levels of the FLS, CHI, and CHS genes were likewise correlated favorably with the phenolic and flavonoid contents. Additionally, the expression levels of the CHS and FLS genes were positively correlated with the growth parameters. Conversely, sand and silt percentages, EC in soil, in addition to total anions, Co_3_, Hco_3_, So_4_, Cl, total cations, Fe, Ca, Na, and Mg, in addition to the fresh and dried biomasses and primary and secondary metabolites in plants, were negatively correlated with MDA and H_2_O_2_ contents. (Fig. 4).

## Discussion

The characterization of Khuzam shale (Djdi) reveals its potential as a multi-nutrient mineral fertilizer, albeit with significant caveats. Its nano-scale particle size (average 72 nm) is a critical attribute, as it confers a high surface area-to-volume ratio, potentially enhancing the solubility and bioavailability of its constituent minerals [34, 35]. The rich composition of essential macro-elements like Ca^+ 2^, Mg^+ 2^, K^+^, and S^+ 2^, alongside micronutrients such as Fe, aligns with the mineral requirements for plant growth [35–37]. The presence of these elements in mineral forms like calcite, dolomite, and anhydrite provides a slow-release nutrient source, which can be beneficial for sustained plant nutrition. However, the exceptionally high salinity of pure Djdi, evidenced by the peak values of total anions/cations, Na^+^, and Cl^-^, positions it as a potent abiotic stressor [38]. The complete inhibition of seed germination in pure Djdi soil directly corroborates this, as high salt concentrations create a low osmotic potential, preventing water uptake and causing ionic toxicity [35, 39]. The significant increase in soil EC from 1.99 ms/cm in the control to 15.18 ms/cm in Djdi treatment further confirms the severe salt stress imposed [12, 40]. Soil pH of all treatments ranged between 7.72 and 7.89; all are basic soils that are more suitable for plant growth [40].

Atomic Absorption Spectrometry analysis revealed that the application of Khuzam shale (Djdi) significantly enhanced the soil’s mineral content. All treated soils (Djdi: CS1, CS2, CS3) showed elevated levels of total anions and cations, including CO₃^-2^, Cl^⁻^, HCO₃^⁻^, SO₄^-²^, K⁺, N^+^, P^+ 5^, Fe^+ 3^, Ca^+^², Na⁺, and Mg²⁺, compared to the control, with the highest residual soil concentrations predominantly found in Djdi: CS2 [37]. This aligns with the established mineral richness of Djdi, as documented by [12, 41]. Paradoxically, despite having lower residual mineral levels in the soil, plants grown in the Djdi: CS3 treatment exhibited the highest tissue concentrations of essential nutrients like N, P, K, and Ca [37]. This suggests that in the Djdi: CS3 formulation, minerals were present in a more bioavailable form rather than as complex or insoluble compounds, facilitating greater plant uptake and assimilation [35, 36]. This optimal bioavailability in Djdi: CS3 likely underpins the superior plant growth and metabolic performance, a conclusion strongly supported by the physiological, genetic, and correlation data [42, 43].

Pure Djdi soil significantly inhibited the growth of *E. vesicaria*, consistent with findings that sediment properties critically influence plant development [44]. The observed growth stunting is likely caused by mineral imbalances, where either excessive level of essential elements creates oxidative stress that inhibits plant development and imposes a physiological burden on vegetative growth. Consequently, oxidative stress was significantly elevated in the pure Djdi soil, as indicated by the elevation of H₂O₂ and MDA. The generation of ROS groups, such as superoxide radical, hydrogen peroxide (H_2_O_2_), and hydroxyl radical, is correlated with the oxidative stress triggered by the trace element contamination. Furthermore, the high metal ion content in the soil can decompose lipid hydroperoxides via hemolytic cleavage of their O-O bond. This reaction generates lipid alkoxyl radicals, which subsequently propagate a chain reaction of free radical oxidation [45]. This aligns with the findings of Alharbi et al., who reported a similar accumulation of MDA and H_2_O_2_ in *Avicennia marina* plants germinated in mineral and oxide-rich soil [46]. This indicates that Djdi’s agricultural value requires balanced integration with other soil components to mitigate its inherent limitations. The synergistic effect of Djdi and CS was clearly demonstrated in the plant responses. The application of Djdi: CS mixtures reversed the germination failure and significantly enhanced seedling growth, fresh weight, and dry weight compared to both the control and pure Djdi treatments. This growth promotion can be attributed to the improved mineral nutrition, particularly the enhanced uptake of P and K, which are vital for energy transfer, photosynthesis, and osmotic regulation [47, 48].

In this regard, the application of Djdi: CS treatments, particularly the Djdi: CS3 formulation, significantly stimulated the biosynthesis of total phenolics and flavonoids in *E. vesicaria*. This marked increase confirms the elicitor potential of the Djdi: CS amendment [36]. These secondary metabolites are potent non-enzymatic antioxidants, known for their ROS-scavenging capacity and their vital roles in plant development and cell wall structure [32, 49]. Critically, the Djdi: CS3 treatment successfully enhanced the phenylpropanoid pathway, leading to a robust accumulation of defensive secondary metabolites without compromising plant growth, indicating a beneficial reallocation of resources from primary to secondary metabolism. Thus, when compared to the control, the alterations in the gene expression level of PAL; the primary enzyme in the phenylpropanoid pathway, amplified dramatically at Djdi: CS3. Consequently, the overexpression of PAL indicated the stimulation of phenylpropanoid pathways, which was proven by the previously described rise in phenolic and flavonoid levels caused by Djdi: CS administration. This was corroborated by the high Pearson correlation (*p* < 0.05) in Fig. 6. Shi et al., showed that the expression of *PAL* in *Vitis vinifera* was overexpressed with lower mineral treatments and downregulated with higher mineral treatments. Further investigation revealed coordinated upregulation of critical flavonoid biosynthesis genes [50]. Moreover, in comparison to the control, Djdi: CS3 treatment led to a notable improvement in the expression of the genes for *FLS*, *CHI*, and *CHS*, which were prospectively associated with their operations and enzyme activities. *CHS* represented the first key enzyme in the synthesis of flavonoids that catalyze the production of chalcone as a central intermediate via the integration of coumaroyl-CoA [51]. It is also known as the first rate-limiting enzyme, where its activation is linked to the operation of the synthesis of flavonoids [51, 52]. The role of the second key rate-limiting enzyme, CHI, was frequently identified to induce the isomerization of chalcone derivatives into direct precursors for several flavonoids such as naringenin and liquiritigenin [53, 54]. Consistent with our findings, in tobacco plants [54] and *A. thaliana* [55], rising *CHI* transcript levels were positively correlated with flavonoid content. In essence, FLS functions as a rate-limiting enzyme in the production of flavonol and is another gene that codes for the primary enzyme in the synthesis of flavonoid metabolites [50, 54]. The hydroxylated flavonoids at position C-3, such as myricetin, quercetin, and kaempferol, are produced when FLS triggers the desaturation of dihydroflavonols [56]. Consequently, the results showed that the Djdj: CS treatments had an intriguing capacity to stimulate secondary metabolic pathways, leading to an increased accumulation of bioactive compounds.

## Conclusion

This study demonstrates the considerable potential of Djdi Ocher (Khuzam Shale), an ancient pharaonic natural fertilizer, as a sustainable method for enhancing soil fertility and agricultural output. Combining Djdi with sandy clay soil in specified proportions, notably Djdi: CS3 (1:3), has shown the most efficacy in improving nutrient content, germination rates, and plant development metrics physiologically and genetically of *E. vesicaria*, while facilitating the uptake of essential elements, including nitrogen, phosphorus, potassium, and iron. Accordingly, this study recommended the future application of Djdi: soil mixtures for the growth of medicinal and other strategic plants, especially under stress conditions such as drought. Additionally, for large-scale applications, we recommend using a crop rotation system in fertilized soil after planting watercress, particularly for nutrient-demanding crops. Mixing Djdi boosts plant growth and enriches the soil for future crops. We intend for future research to focus on modelling the dose-response relationship using a broader range of intermediate Djdi-to-soil ratios to identify the precise optimal concentration and the mechanisms of its stimulatory and inhibitory effects. 

## Supplementary Information

Supplementary Material 1.

## Data Availability

The manuscript contains all the data that substantiates the findings of this investigation. All data is available by request from the corresponding author.

## Abbreviations

Djdi: Khuzam Shale
XRD: X-ray diffraction
pH: Potential of hydrogen
EC: Electrical conductivity
AAS: Atomic Absorption Spectroscopy
APX: Ascorbate peroxidase
PAL: Phenylalanine ammonia lyase
CHS: Chalcone synthase
FLS: Flavonol synthase
CHI: Chalcone isomerase
H_2_O_2_: Hydrogen peroxide
MDA: Malondialdehyde
ANOVA: Analysis of Variance
